# Applicable safety analysis and biomechanical study of iliosacral triangular osteosynthesis

**DOI:** 10.1186/s12891-021-04856-8

**Published:** 2021-11-23

**Authors:** Jianxiong Zheng, Jie Xiang, Xiaoreng Feng, Fei Liu, Keyu Chen, Bin Chen

**Affiliations:** 1 Division of Orthopaedics and Traumatology, Department of Orthopaedics, Nanfang Hospital, Southern Medical University, No. 1838 North Guangzhou Avenue, Guangzhou, 510515 China; 2 Department of Orthopaedics and Traumatology, the First Affiliated Hospital of University of South China, Hengyang, China; 3 Department of Orthopaedics and Traumatology, Yangjiang People’s Hospital, Yangjiang, China

**Keywords:** S2-alar-iliac screw, Triangular osteosynthesis, Sacral fracture, Iliosacral fixation, Biomechanics

## Abstract

**Background:**

The aim of this study was to investigate the applicable safety and biomechanical stability of iliosacral triangular osteosynthesis (ITO) through 3D modeling and finite element (FE) analysis.

**Methods:**

Pelvic CT imaging data from 100 cases were imported into Mimics software for the construction of 3D pelvic models. The S2-alar-iliac (S2AI) screws and S2 sacroiliac screws were placed in the S2 segment with optimal distribution and their compatibility rate on the S2 safe channel was observed and analyzed. In the FE model, the posterior pelvic ring was fixed with two transsacral screws (TTS), triangular osteosynthesis (TO) and ITO, respectively. Four different loading methods were implemented in sequence to simulate the force in standing, flexion, right bending, and left twisting, respectively. The relative displacement and change in relative displacement of the three fixing methods were recorded and analyzed.

**Results:**

The theoretical compatibility rate of S2AI screw and S2 sacroiliac screw in S2 segment was 94%, of which 100% were in males and 88% in females. In the FE model, in terms of overall relative displacement, TTS group showed the smallest relative displacement, the ITO group showed the second smallest, and the TO group the largest relative displacement. The change in relative displacement of the TTS group displayed the smaller fluctuations in motion. The change in relative displacement of the TO group under right bending and left twisting displayed larger fluctuations, while the ITO group under flexion displayed larger fluctuations.

**Conclusions:**

The simultaneous placement of S2AI screw and S2 sacroiliac screw in the S2 segment is theoretically safe. Although the biomechanical stability of ITO is slightly lower than TTS, it is better than TO, and can be used as a new method for the treatment of posterior pelvic ring injuries.

**Supplementary Information:**

The online version contains supplementary material available at 10.1186/s12891-021-04856-8.

## Background

Vertical shear pelvic fracture is an unstable injury caused by the complete rupture of the anterior and posterior pelvic ring [1]. Due to complex local anatomy, unique biomechanics and poor bone quality, the fixation of sacral fractures remains a challenge [2]. A key aspect of posterior pelvic ring injury repair is to have sufficient stability to balance stress in the vertical and horizontal direction [3]. Single horizontal fixation, such as sacral rod, sacroiliac screw, tension band plate, local plate, and transiliac internal fixator, or vertical fixation (unilateral lumbopelvic fixation, LPF) do not fully meet the above requirements [4–7].

Schildhauer et al. [3] first proposed the concept of triangular osteosynthesis (TO), which combines vertical fixation (unilateral LPF) and horizontal fixation (sacroiliac screw) for the treatment of sacral fractures. Biomechanical studies have shown that TO provides higher stability than a sacroiliac screw, tension band plate, and unilateral LPF [7–9]. Although TO has been widely used in clinical practice, the following limitations still exist. First, the protruding iliac screws often cause local pain and even pressure sores to patients [4]. Second, LPF restricts normal lumbosacral joint activities and long-term placement can easily cause low back pain and lumbosacral scoliosis [10]. Additionally, implants often need to be surgically removed. Finally, extensive exposure of incisions increases the incidence of surgical site infections, especially in patients with multiple traumas [11]. Although modified designs for the TO to avoid fixing the lumbar spine have been proposed, problems caused by the protrusion of the iliac screw cannot be avoided [12, 13].

Transsacral screw fixation has been considered as an alternative to sacroiliac screw fixation, and has been effectively used in remedying the failure of sacroiliac screw fixation [14, 15]. Biomechanical studies have shown that two transsacral screws (TTS) provide higher stability than sacroiliac screws and TO [16, 17]. However, only 63% of men and 66% of women have the S1 transsacral screw channel, while 100% of men and 87% of women have the S2 transsacral screw channel [18]. Therefore, the variation of the sacrum limits the application of TTS.

Recently, Lee et al. [19] proposed the S2-alar-iliac (S2AI) screw and the S1 pedicle screw fixation (S2AI-S1) and added an S1 sacroiliac screw as a new improvement in TO. Compared with TO, this improved fixation method may not only avoid the complications of lumbar fixation, but also reduce the risk of screw protrusion. However, only one case was used in clinical practice and the safety and biomechanical properties of the proposed methods were not further studied. In addition, some variants of the sacrum reduce the S1 safe channel, while the S2 safe channel increases [20, 21]. Therefore, there may not be enough safe space to place two screws in the S1 segment for the variant sacrum.

We propose the combination of S2AI-S1 fixation with an S2 sacroiliac screw (i.e., iliosacral triangular osteosynthesis, ITO) for the treatment of sacral fractures. However, it is not yet clear whether simultaneous placement of two screws (i.e., S2AI screw and S2 sacroiliac screw) in the S2 segment is feasible and the biomechanical properties of ITO are also unclear.

The aim of this study was to investigate the applicable safety and biomechanical stability of ITO through 3D modeling and finite element (FE) analysis. This study was approved by the Ethics Committee of the Nanfang Hospital of the Southern Medical University (NFEC-2019-256). Informed consent was obtained from all participants included in the study.

## Materials and methods

### 3D modeling and measurement

The pelvic CT data of 100 patients (50 males and 50 females, aged between 18 and 60, with normal bone structure and no damage) from the Nanfang Hospital were saved in DICOM format and then were imported into Mimics 21.0 (Materialise, Leuven, Belgium) to reconstruct pelvis model. The transparency of the sacrum and ilium were adjusted in the software to show the S2 safety channel in the lateral view. A tangent line along the upper wall of the S2 safety channel and a line parallel to the upper tangent line through the lowest point of the S2 safety channel were drawn. In this study, the vertical distance between the two parallel lines was defined as the upper and lower width of the S2 safety channel.

**Fig. 1:**
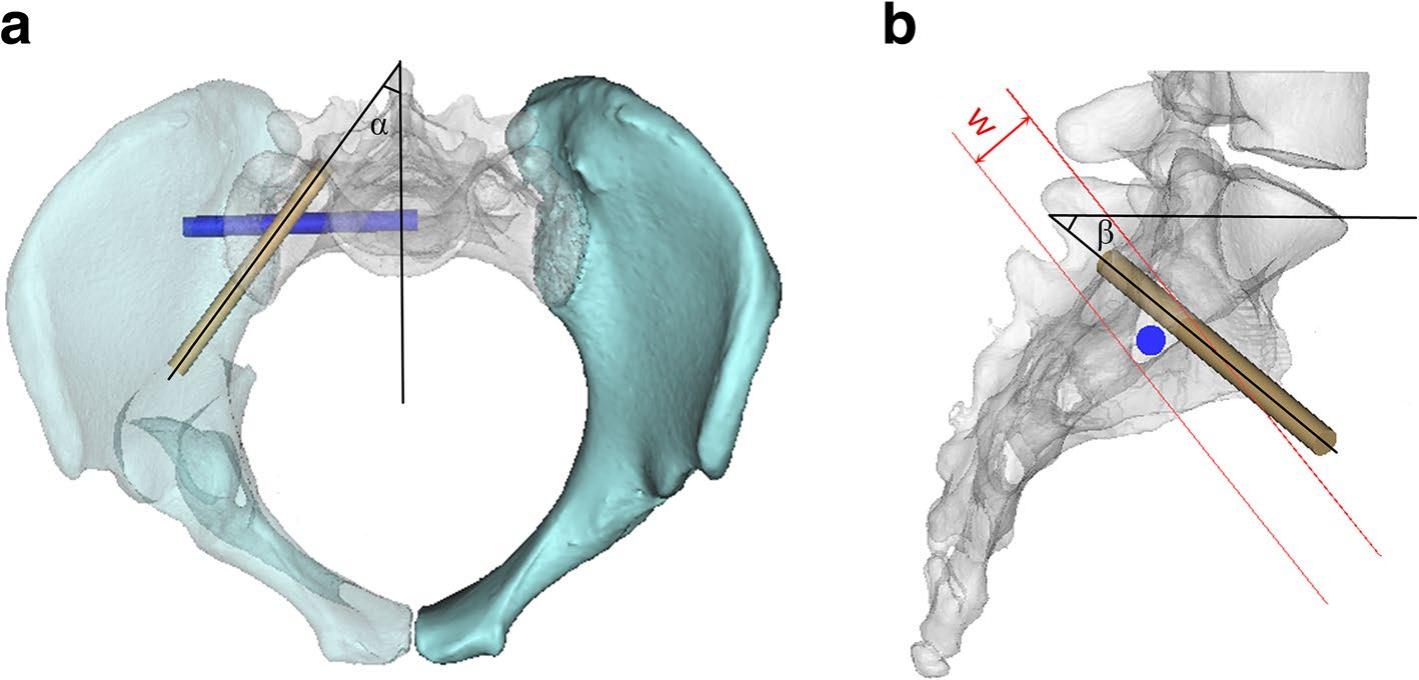
Establishment of 3D pelvic model and placement of S2AI screw (grey) and S2 sacroiliac screw (blue) in S2 segment. **a**. The inlet view of the pelvis. The projection of the S2AI screw on the horizontal plane is used to measure the lateral angulation(α). **b**. The lateral view of the pelvis. The projection of the S2AI screw on the sagittal plane is used to measure the caudal angulation(β). The W shown in the figure is the upper and lower width of the S2 safety channel

According to the literature, the S2AI screw (diameter 7.0 mm, length 90 mm) was placed 1-mm inferior and 1-mm lateral to the S1 dorsal foramen and the trajectory for the S2AI screw was aimed at the anterior inferior iliac spine [22]. Under the prerequisite of ensuring safety of the S2AI screw, the S2AI screw lateral angulation and caudal angulations were appropriately adjusted to leave as much space as possible for the S2 sacroiliac screw (optimal distribution). An S2 sacroiliac screw (diameter 7.0 mm, length 80 mm) was then placed in the horizontal direction and finally we observed whether the S2 sacroiliac screw would penetrate the S2 safety channel. In this study, compatibility was used to indicate that the S2 segment can accommodate the S2AI screw and S2 sacroiliac screw, that is, the S2AI screw was placed without breaking through the medial or lateral iliac cortices, and the S2 sacroiliac screw did not penetrate the S2 safety channel. The lateral angulation and caudal angulation of S2AI screws were measured in all compatible cases (Fig. 1a-b).

Statistical analyses were performed using SPSS 20.0 software (IBM Corp., Armonk, NY, USA) and the Student’s t test was used to compare continuous variables. Data are shown as the mean ± standard deviation of the mean. *P*<0.05 indicated statistical significance.

### Finite element analysis

CT data of the lumbar spine and pelvis were collected from a healthy male volunteer (30 years old, 175 cm, 70 kg, normal bone structure, no tumors, no deformities, no lumbar spine and pelvic structural damage). The CT imaging data were processed by Mimics 21.0 (Materialise, Leuven, Belgium), Geomagic Studio 2013 (Geomagic, Morrisville, NC, USA), and Solidworks 2017 (Dassault Systèmes Corp., Vélizy-Villacoublay, France) for the reconstruction of a model with lumbar spine and complete pelvis. The interpelvic ligaments were simulated as spring structures in finite element (FE) analysis software, ANSYS 17.0 (ANSYS Inc., Canonsburg, PA, USA). The Young’s modulus (MPa) and Poisson’s ratio (u) were assigned as mechanical properties to the cortical bone, cancellous bone, articular cartilage, intervertebral disc, interpubic disc, titanium metal, with values that were obtained from the literature [23, 24]. The properties of ligaments were expressed in stiffness (N/mm) [25]. According to the literature, the thickness of the cortical bone is 1.3 mm and the thickness of the endplate is 0.8 mm [8]; the thickness of sacral cartilage is 1.8 mm, the thickness of ilium cartilage is 0.9 mm, and the distance between the two is 0.3 mm [26]. All bony parts and implants were meshed using a 10-node tetrahedron element. The FE model of the intact pelvis had 459,703 nodes and 258,368 elements (Table 1).

**Table 1:** The material properties used in the finite element models

Materials	Young’s modulus (MPa)	Poisson’s ratio(u)	Reference	ligament	Stiffness (N/mm)	Number of elements	Reference
Titanium screw/rod/plate	110,000	0.3	[24]	Anterior sacroiliac ligament	700	10 × 2	[25]
Cortical bone (Pelvis)	17,000	0.3	[23]	Posterior sacroiliac ligament (long)	1000	4 × 2	[25]
Cancellous bone (Pelvis)	100	0.2	[23]	Posterior sacroiliac ligament (short)	400	10 × 2	[25]
Cortical bone (Lumbar)	12,000	0.3	[23]	Interosseous sacroiliac ligament	2800	4 × 2	[25]
Cancellous bone (Lumbar)	100	0.2	[23]	Sacrospinous ligament	1400	5 × 2	[25]
Posterior bony elements	3500	0.25	[24]	Sacrotuberous ligament	1500	5 × 2	[25]
Articular cartilage	10	0.4	[24]	Superior pubic ligament	500	1 × 1	[25]
Bony endplate	1000	0.4	[24]	Accurate pubic ligament	500	1 × 1	[25]
Cartilage endplate	25	0.25	[24]	Iliolumbar ligament	1000	4 × 2	[25]
Nucleus pulposus	1	0.499	[24]				
Matrix of annulus fibrosus	4.2	0.45	[24]				
Fibers of annulus fibrosus	450	0.3	[24]				
Interpubic disc	5	0.45	[24]				

**Fig. 2:**
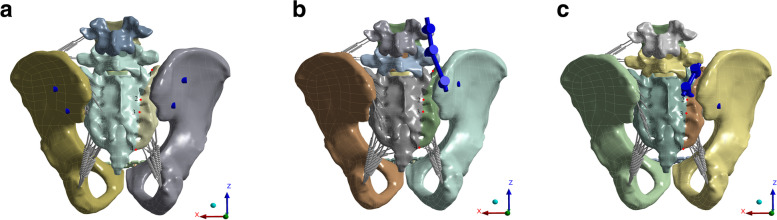
Illustration of different internal fixation methods for Denis type II sacral fractures. **a**. Two transsacral screws (TTS) group model. **b**. Triangular osteosynthesis (TO) group model.c.Iliosacral triangular osteosynthesis (ITO) group model. The picture shows α4 pairs of different observation points (red dots) from top to bottom behind the sacrum

The Denis type II sacral fracture injury model was constructed by grid line segmentation and the ligament was also broken when the fracture occurred. Some ligaments on the right side of the pelvis were removed from the model, including the anterior sacroiliac ligament, the long posterior sacroiliac ligament, the iliolumbar ligament, part of the sacrospinous ligament, and part of the sacrotuberous ligament. Subsequently, the interpubic disc and the corresponding superior pubic ligament and arcuate pubic ligament were removed to complete the construction of the Tile C pelvic injury model. In all models, the anterior pelvic ring was fixed with a plate, and the posterior pelvic ring was fixed with TTS, TO, and ITO, respectively. The TTS model consisted of one S1 transsacral screw and one S2 transsacral screw. The TO model consisted of a unilateral LPF and an S1 sacroiliac screw. The ITO model consisted of a S2AI-S1 and an S2 sacroiliac screw. Iliac screws/S2AI screws, S1/S2 transsacral screws and S1/S2 sacroiliac screws were all fully threaded screws with a diameter of 7.0 mm. The length of the ilium screw/S2AI screw was 80 mm, and the length of the S1/S2 sacroiliac screw was 75 mm (Fig. 2a-c).

The contact conditions of the interaction surfaces of the sacroiliac joints and the fracture interaction surfaces were set to friction, with the corresponding friction coefficients set as 0.015 and 0.3, respectively [27]. The interfaces between the superior and inferior articular processes, the intervertebral disc/pubic disc/articular cartilage and bone, the plates/bars and the screws, and the screws and bone were modeled with a bonded contact.

A posture of standing with two legs was simulated: the acetabulum on the two sides was fixed in six degrees of freedom. A 500 N vertical stress was applied to the upper surface of the L4 vertebral body to simulate the physiological load of the body above the waist. In addition, an additional 10 N·M torque was applied in three different directions to simulate flexion, right bending and left twisting movement, respectively.

Four pairs of observation points from top to bottom on both sides of the sacral fracture line were selected (Fig. 2a-c). The relative displacements of the four pairs of observation points under the standing, flexion, right bending, and left twisting motions were calculated. The relative displacement was used to evaluate the effect of internal fixation on the overall stability of the fracture model, with low relative displacement pointing to better fixation stability. In addition, to evaluate the relative displacement fluctuation range of the motion conditions relative to the standing conditions the change in relative displacement of the three types of motions with respect to standing were calculated **(**Additional file 1**)**.

## Results

### Measurement result

The upper and lower width of the male S2 safe channel was 14.82 ± 1.81 mm (10.27–18.66 mm) and the upper and lower width of the female S2 safe channel was 13.84 ± 2.14 mm (8.17–17.33 mm) (*P* = 0.015). This indicates that the upper and lower width of the S2 safety channel is significantly larger in men, rather than in women. Except for 2 patients (both female) who did not have the S2 safety channel, 94 patients were able to accommodate the S2AI screw and S2 sacroiliac screw in the S2 segment, and 4 patients (all female) could not accommodate S2AI screw and S2 sacroiliac screw in the S2 segment. The theoretical overall compatibility rate of S2AI screws and S2 sacroiliac screws in the S2 segment was 94%, of which 100% were males and 88% females. The lateral angulation of the S2AI screw in males was 39.78° ± 4.00° and 41.90° ± 3.79° in females (*P* = 0.01). These findings indicated that the lateral angulation of S2AI screw in women was significantly larger compared to that in man. The caudal angulation of the S2AI screw in males was 32.69° ± 3.67° and 33.99° ± 3.90° in females (*P* = 0.10).

### Finite element analysis results

Validation of the pelvic FE models: The maximum compressive displacement (0.640 to 1.136 mm) predicted from the FE analysis of the intact pelvic model under 500 N vertical load was consistent with the corresponding experimentally measured peak compressive displacements (0.973 to 1.550 mm) reported by Comstock et al. [28] under the same loading condition. In addition, according to the experimental conditions of Yongtao Lu et al. [8], a 6.5 mm sacroiliac screw was used to fix the Tile C type pelvic ring injury. The average displacement of the observation site of the sacral wing edge was 1.582 mm, which was close to the 2.0 mm displacement reported in the literature. Hip bones were positioned upside down under similar experimental conditions as reported by Dalstra et al. [29], and the FE model predicted von Mises stresses (3.259 to 10.747 MPa under 600 N loads) of a hip-bone material was consistent with the corresponding experiment-measured von Mises stresses at the eight strain gages (0.712 to 7.641 MPa under 600 N loads).

**Fig. 3:**
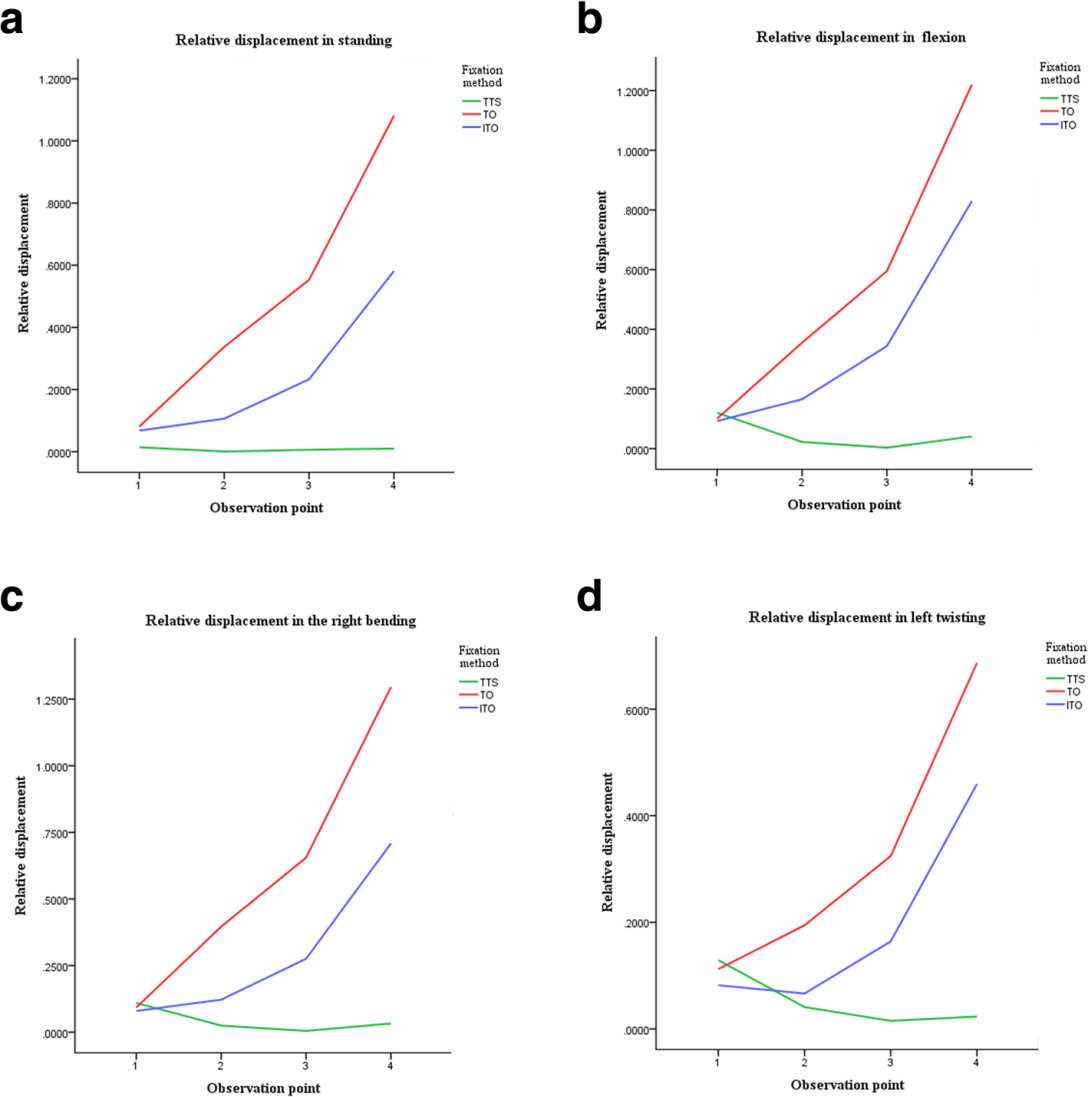
The relative displacement distribution diagrams in four loading conditions. **a** .The relative displacement in standing. **b**. The relative displacement in flexion. **c**. The relative displacement in right bending. **d**. The relative displacement in left twisting. TTS: Two transsacral screws; TO: Triangular osteosynthesis; ITO: Iliosacral triangular osteosynthesis

Relative displacement under four loading conditions **(**Additional files 2, 3, 4 and 5**)**: From observation point 1 to 4, the relative displacement of the TO and ITO groups showed a gradual increasing trend, with the displacement of the former being greater than that of the latter. For the TTS group, the relative displacement was significantly lower than the two other groups, except at observation point 1, at which all groups were close. The relative displacement of the TTS group was less affected by movement. The relative displacement of the TO and ITO groups was increased under flexion and right bending, compared to standing, and the relative displacement was reduced under left twisting, compared to standing (Fig. 3a-d).

The change in relative displacement of the three types of movement (compared to standing): Τhe change in relative displacement of the TTS group at the other observation points was lower than that of the TO and the ITO groups, except at observation point 1, where the displacement change of the TTS group was slightly larger than the other two groups. The change in relative displacement of the TO group under right bending and left twisting was larger than that of the ITO group and the change in relative displacement under flexion was smaller than that of the ITO group (Table 2).

**Table 2:** The change in relative displacement of the three fixation methods

Motion	Fixation method	Relative displacement (mm)			
		1	2	3	4
Flexion-standing	TTS	0.1061	0.0229	0.0067	0.0310
	TO	0.0313	0.0547	0.0706	0.1627
	ITO	0.0250	0.0623	0.1152	0.2539
Right bending- standing	TTS	0.0958	0.0250	0.0070	0.0227
	TO	0.0309	0.0674	0.1078	0.2167
	ITO	0.0119	0.0164	0.0430	0.1281
Left twisting-standing	TTS	0.1150	0.0411	0.0205	0.0129
	TO	0.0476	0.1924	0.2717	0.4376
	ITO	0.0140	0.0433	0.0718	0.1240

*TTS* Two transsacral screws, *TO* Triangular osteosynthesis, *ITO* Iliosacral triangular osteosynthesis

## Discussion

Several past literature studies have measured the safe passage of S2 sacroiliac screws and S2AI screws, also applying 3D-CT technology [18, 21, 22, 30–32]. However, there is a lack of literature investigating a possible simultaneous placement of the two screws in the S2 segment. Due to the changes in the lateral angulation and caudal angulation of the S2AI screw [22], some uncertainty exists on the safe insertion of the S2 sacroiliac screw. To clarify the factors affecting the safe placement of the two screws into the S2 segment, the placement of two screws was simulated in an optimized manner on the 3D pelvic model qualitatively. Studies have shown that the theoretical male compatibility rate is 100%, while in females it reaches 88%. According to the characteristic that the S2AI screw and S2 sacroiliac screw are arranged up and down in the lateral view, the upper and lower width of the S2 safety channel in the lateral view was used as the quantitative analysis standard. Previous studies have shown that the normal pelvic S2 safety channel is often smaller than S1, so the risk of S2 sacroiliac screw placement may be relatively higher [18]. However, in the case of dysmorphic sacrums, a study showed that the S2 segment provides a larger safety channel for screw insertion than S1 and insertion of the significantly longer screws is possible in S2, compared with the dysmorphic S1 segment [20]. Furthermore, another study found that the S2 axial transverse widths (i.e., the upper and lower widths) of the variant sacrum are larger than those of the normal sacrum [21]. This indicates that it may be more beneficial to the use of ITO for patients with variant sacrum.

The factors that affect the compatibility of S2AI screws and S2 sacroiliac screws are summarized into the following points: The first is regarding to the upper and lower width of the S2 safety channel. In theory, the larger the upper and lower width of the S2 safety channel is, the larger the safety channel is. In this study, we showed that when the upper and lower width of the S2 safety channel is less than 12 mm, the risk of the S2 sacroiliac screw passing through the S2 safety channel increases. To ensure safe insertion of the two screws, we recommend the upper and lower width of the S2 safety channel to be 12 mm as the minimum clinically applicable standard for ITO. The second factor concerns the shape of the S2 safety channel. Most individuals (92%) included in the study showed an approximately fan-shaped section of the S2 safety channel, while few showed an approximately triangular (3%) or trapezoidal section (5%). When the length of the upper wall and the front wall of the safety channel is constant, the area of the trapezoidal section is the largest (i.e., the most favorable), the sector section is the second largest and the area of the triangular section is the smallest (i.e., the most unfavorable). The final factor is the caudal angulation and the screw diameter of the S2AI screw. The larger the caudal angulation and diameter of the S2AI screw are, the larger the safe channel section of the S2 sacroiliac screw occupied by the S2AI screw is. Therefore, the S2AI screw diameter is recommended not to exceed 7.0 mm. In this study, the average caudal angulation of females was slightly smaller than the results presented by Zhu et al. [22]. This may be caused by adjusting the trajectory of the S2AI screw to reserve a safe space for the S2 sacroiliac screw.

Min et al. [16] compared the fixation effect of two transsacral screws both in the S1 segment and TO on Denis type II sacral fractures in a biomechanical study. The study showed that the vertical stability of the two transsacral screws was higher in comparison to the TO. In our study, the distribution of the two transsacral screws in the TTS group was slightly different from that presented by Min et al., however, the results of this study suggested that TTS was more stable than TO for Denis II sacral fractures, which was consistent with the data presented by Min et al. It is postulated that the stability in TTS comes from the biplanar stability of two screws and the additional cortical purchase in the contralateral ilium [16]. Shannon et al. [15] further confirmed that the stability of full-threaded screws in TTS is better than the stability of half-threaded screws. It is, therefore, reasonable to conclude that the above three factors are the source of stability of TTS in this study. The stability of ITO was better than that of TO in this study. In a biomechanical study, after 5000 cycles of loading of the pelvic model, the relative displacement of S2AI-S1 was found to be significantly smaller than that of unilateral LPF. It is evident from the data that, the main reason for the higher stability of ITO is that S2AI-S1 is more stable than unilateral LPF. In addition, Zhao et al. [17] confirmed that the stability of an S2 sacroiliac screw is slightly better than that of an S1 sacroiliac screw.

Hu et al. [27] found that the vertical displacement trend under flexion and right bending is the same as during standing, which is also confirmed in this study. However, the vertical displacement cannot accurately reflect the effect of additional stress on the model. In this study, the change in relative displacement was used as the feedback to the additional applied torque of 10 N·M. The study showed that the change in relative displacement of the two transsacral screws was the smallest under the three motions, except in observation point 1, which further explains the stability of the TTS group. The change in relative displacement of ITO was larger than TO under flexion and smaller under right bending and left twisting. This indicates that flexion will relatively increase the instability of ITO, while right bending and left twisting will relatively increase the instability of TO.

Percutaneous transsacral screw placement has the advantages of less damage, better biomechanical stability, and fewer postoperative complications, making it widely used in clinical practice [14, 33, 34]. However, due to the complex anatomy of the posterior pelvic ring and the variability of the sacrum, the difficulty and risk for transsacral screw placement is much higher than standard sacroiliac screws [33, 34]. Compared with TO, ITO is only fixed on the sacrum, so it does not affect the normal lumbosacral activities of the patient. In addition, ITO may reduce the incidence of low back pain, lumbosacral scoliosis, and incision infection caused by TO [10, 12, 19]. Furthermore, the S2AI screw is 15 mm deeper than the insertion point of the iliac screw [31], which may reduce the local pain caused by the protrusion of the implant. The results of this study confirmed that the overall biomechanical stability of ITO is better than that of TO. Nevertheless, ITO is not suitable for all patients. In addition to the preoperative measurement of the S2 safety channel being not compatible with the two screws, ITO is not recommended for specific fracture types, such as S1 vertebral comminuted fracture, lumbosacral instability, or posterior iliac wing fracture. The possibility of the use of ITO is can only be determined by excluding all the influencing factors that interfere with the placement of screws. For patients with unilateral compromise of the S1 pedicle, which makes screw placement difficult or impossible, connecting the L5 screw and S2AI screw will be a good alternative.

### Limitations

This study used the undamaged sacral S2 safety channel as a qualitative analysis model, but anatomical reduction is not always achieved in clinical practice. In addition, the S2AI screw and S2 sacroiliac screw may not be optimally distributed on the pelvis. Therefore, the compatibility rate of S2AI screws and S2 sacroiliac screws obtained in this study may be slightly different from the clinical results. Additionally, only the main ligaments were simulated in the FE analysis, but in reality, other ligaments and muscles may also have an effect [35]. Therefore, there may be some differences between our simulative data and the cadaver bone model data. Thirdly, the simulation results were based on static linear displacement analysis, and the analysis of screw loosening and failure behavior was not considered. This would need to be further verified by cyclic loading experiments on cadaver bone models. Finally, this study used CT data of a normal male pelvis for the FE model development, and no anatomical differences between individuals were considered.

## Conclusions

The simultaneous placement of S2AI screw and S2 sacroiliac screw in the S2 segment is theoretically safe. Although the biomechanical stability of iliosacral triangular osteosynthesis is slightly lower than two transsacral screws, it is better than triangular osteosynthesis, and can be used as a new method for the treatment of posterior pelvic ring injuries.

## Supplementary Information

**Additional file 1.**

**Additional file 2.**

**Additional file 3.**

**Additional file 4.**

**Additional file 5.**

## Data Availability

All data generated or analysed during this study are included in this published article and its supplementary information files.

## Abbreviations

FE: Finite element
S2AI: S2-alar-iliac
ITO: Iliosacral triangular osteosynthesis
TO: Triangular osteosynthesis
TTS: Two transsacral screws
LPF: Lumbopelvic fixation
S2AI-S1: S2-alar-iliac screw and S1 pedicle screw fixation
