# Supplementary material for: Applicable safety analysis and biomechanical study of iliosacral triangular osteosynthesis
Source: BMC Musculoskelet Disord. 2021 Nov 23;22:971. doi: 10.1186/s12891-021-04856-8 (PMC8609831; doi:10.1186/s12891-021-04856-8)
Supplement: Supplementary file 1 — Additional file 1. [file 12891_2021_4856_MOESM1_ESM.pdf]

Additional file 1

The calculation formula of relative displacement and relative displacement change in this study

1. Mark the observation point on the inner side of the fracture line as 1a, and mark the observation point on the outer side of the fracture line as 1b.
2. After 500N vertical loading, point 1 is displaced on the X, Y, and Z axes respectively. The displacement of point 1a (the positive and negative signs represent different displacement directions) is marked as (X1a.Y1a.Z1a), and the coordinates of point 1b are marked as (X1b.Y1b.Z1b).
3. Therefore, the calculation formula for the relative displacement (RD) of position 1 in the standing is:RD= $\sqrt{[(X1_a - X1_b)^2 + (Y1_a - Y1_b)^2 + (Z1_a - Z1_b)^2]}$
4. In the flexion movement , point 1 is displaced again on the X, Y and Z axes respectively, the coordinate of point 1a is changed and the mark is 1a' (X1a'.Y1a'.Z1a'), and the coordinate of point 1b is changed. It is 1b' (X1b'.Y1b'.Z1b'). Compared with standing, the relative displacement of the medial point of the fracture line in space is marked as A (X1a'- X1a. Y1a'- Y1a. Z1a'- Z1a), and the relative displacement of the corresponding outer point of the fracture line in space is marked as B (X1b'- X1b. Y1b'- Y1b. Z1b'- Z1b).

5. The calculation formula for the relative displacement change (△RD) of flexion relative to standing at point 1 is:

$$\triangle \text{ RD} = \sqrt{[(XA - XB)^2 + (YA - YB)^2 + (ZA - ZB)^2]}$$

Expanding the above formula is: $\triangle$RD

$$= \sqrt{[(X1b' - X1b) - (X1a' - X1a)]^2 + [(Y1b' - Y1b) - (Y1a' - Y1a)]^2 + [(Z1b' - Z1b) - (Z1a' - Z1a)]^2}$$

6. By analogy, calculate the RD and △RD of other points respectively.

Note: The RD and △RD methods are different, and the actual meanings they represent are also different. The former represents the total displacement of a point, and the latter represents an increment or decrement. When the RD and △RD are both small, it can better show that this kind of fixing method has the best stability performance.
