# Supplementary material for: Applicable safety analysis and biomechanical study of iliosacral triangular osteosynthesis
Source: BMC Musculoskelet Disord. 2021 Nov 23;22:971. doi: 10.1186/s12891-021-04856-8 (PMC8609831; doi:10.1186/s12891-021-04856-8)
Supplement: Supplementary file 2 — Additional file 2. [file 12891_2021_4856_MOESM2_ESM.pdf]

Additional file 2

Relative displacement in standing

**TTS**	1	2	3	4
Xa<br>Xb	-0.0232<br>-0.0237	-0.0293<br>-0.0297	-0.0275<br>-0.0286	-0.0103<br>-0.0099
RDx(leftward)	0.0005	0.0004	0.0011	-0.0004
Ya<br>Yb	1.3590<br>1.3727	0.9211<br>0.9207	0.7004<br>0.6943	0.0398<br>0.0299
RDy(backward)	-0.0137	0.0004	0.0061	0.0099
Za<br>Zb	-0.7644<br>-0.7607	-1.1053<br>-1.1050	-1.2412<br>-1.2413	-1.2219<br>-1.2215
RDz(upward)	-0.0037	-0.0003	0.0001	-0.0004
**RD**	**0.0142**	**0.0006**	**0.0062**	**0.0099**
**TO**	1	2	3	4
Xa<br>Xb	0.1681<br>0.1577	0.2827<br>0.0481	0.3613<br>-0.0251	0.4790<br>-0.3004
RDx(leftward)	0.0104	0.2346	0.3864	0.7794
Ya<br>Yb	1.5114<br>1.5573	1.2742<br>1.0881	1.1740<br>0.8602	0.8567<br>0.1595
RDy(backward)	-0.0459	0.1861	0.3138	0.6972
Za<br>Zb	-1.0862<br>-1.0206	-1.2672<br>-1.4222	-1.3269<br>-1.5677	-1.3071<br>-1.5809
RDz(upward)	-0.0656	0.1550	0.2408	0.2738
**RD**	**0.0807**	**0.3372**	**0.5530**	**1.0810**
**ITO**	1	2	3	4
Xa<br>Xb	0.0793<br>0.0717	0.0998<br>0.0574	0.1293<br>0.0232	0.1774<br>-0.1723
RDx(leftward)	0.0076	0.0424	0.1061	0.3497
Ya<br>Yb	1.3887<br>1.4384	1.0861<br>1.0090	0.9481<br>0.7835	0.5293<br>0.0913
RDy(backward)	-0.0497	0.0771	0.1646	0.4380
Za<br>Zb	-0.9943<br>-0.9489	-1.2428<br>-1.3024	-1.3279<br>-1.4539	-1.3139<br>-1.4684
RDz(upward)	-0.0454	0.0596	0.1260	0.1545
**RD**	**0.0677**	**0.1063**	**0.2329**	**0.5814**

Point a is located inside the fracture line, and point b is located outside the fracture line. Xa and Xb respectively represent the displacement of the two points

relative to the origin on the X axis. Ya and Yb respectively represent the displacement of the two points on the Y axis relative to the origin. Za and Zb respectively represent the displacement of the two points on the Z axis relative to the origin.

TTS: Two transsacral screws;

TO: Triangular osteosynthesis;

ITO: Iliosacral triangular osteosynthesis

RDx: The relative displacement of the two points a, b on the X axis. Leftward is a positive value

RDy: The relative displacement of the two points a, b on the Y axis. Backward is a positive value

RDz: The relative displacement of the two points a, b on the Z axis .Upward is a positive value

RD: The total relative displacement of two points a, b in the three-dimensional direction
