# Supplementary material for: Applicable safety analysis and biomechanical study of iliosacral triangular osteosynthesis
Source: BMC Musculoskelet Disord. 2021 Nov 23;22:971. doi: 10.1186/s12891-021-04856-8 (PMC8609831; doi:10.1186/s12891-021-04856-8)
Supplement: Supplementary file 3 — Additional file 3. [file 12891_2021_4856_MOESM3_ESM.pdf]

Additional file 3

Relative displacement in flexion

**TTS**	1	2	3	4
Xa<br>Xb	0.0094<br>0.0019	0.0046<br>0.0038	0.0152<br>0.0153	0.0504<br>0.0518
RDx(leftward)	0.0075	0.0008	-0.0001	-0.0014
Ya<br>Yb	-0.0658<br>0.0468	0.0149<br>0.0372	0.0414<br>0.0409	0.1083<br>0.0674
RDy(backward)	-0.1126	-0.0223	0.0005	0.0409
Za<br>Zb	-0.2625<br>-0.2211	-0.2187<br>-0.2151	-0.2038<br>-0.2073	-0.2016<br>-0.2017
RDz(upward)	-0.0414	-0.0036	0.0035	0.0001
**RD**	**0.1202**	**0.0226**	**0.0035**	**0.0409**
**TO**	1	2	3	4
Xa<br>Xb	0.2364<br>0.2220	0.3233<br>0.0722	0.4039<br>-0.0292	0.5232<br>-0.4085
RDx(leftward)	0.0144	0.2511	0.4331	0.9317
Ya<br>Yb	0.2543<br>0.2878	0.4759<br>0.2536	0.6011<br>0.2481	0.9542<br>0.2062
RDy(backward)	-0.0335	0.2223	0.3530	0.7480
Za<br>Zb	-0.6271<br>-0.5330	-0.4977<br>-0.6152	-0.4206<br>-0.6259	-0.4202<br>-0.6677
RDz(upward)	-0.0941	0.1175	0.2053	0.2475
**RD**	**0.1009**	**0.3554**	**0.5953**	**1.2202**
**ITO**	1	2	3	4
Xa<br>Xb	0.1359<br>0.1263	0.1726<br>0.1220	0.2106<br>0.0823	0.2526<br>-0.1982
RDx(leftward)	0.0096	0.0506	0.1283	0.4508
Ya<br>Yb	0.1470<br>0.2168	0.3208<br>0.2075	0.4306<br>0.1891	0.7460<br>0.0949
RDy(backward)	-0.0698	0.1133	0.2415	0.6511
Za<br>Zb	-0.5481<br>-0.4879	-0.4139<br>-0.5235	-0.3475<br>-0.5563	-0.3552<br>-0.6036
RDz(upward)	-0.0602	0.1096	0.2088	0.2484
**RD**	**0.0927**	**0.1656**	**0.3441**	**0.8300**

Point a is located inside the fracture line, and point b is located outside the fracture line. Xa and Xb respectively represent the displacement of the two points relative to

the origin on the X axis. Ya and Yb respectively represent the displacement of the two points on the Y axis relative to the origin. Za and Zb respectively represent the displacement of the two points on the Z axis relative to the origin.

TTS: Two transsacral screws;

TO: Triangular osteosynthesis;

ITO: Iliosacral triangular osteosynthesis

RDx: The relative displacement of the two points a, b on the X axis. Leftward is a positive value

RDy: The relative displacement of the two points a, b on the Y axis. Backward is a positive value

RDz: The relative displacement of the two points a, b on the Z axis .Upward is a positive value

RD: The total relative displacement of two points a, b in the three-dimensional direction
