# Supplementary material for: Applicable safety analysis and biomechanical study of iliosacral triangular osteosynthesis
Source: BMC Musculoskelet Disord. 2021 Nov 23;22:971. doi: 10.1186/s12891-021-04856-8 (PMC8609831; doi:10.1186/s12891-021-04856-8)
Supplement: Supplementary file 4 — Additional file 4. [file 12891_2021_4856_MOESM4_ESM.pdf]

Additional file 4

Relative displacement in right bending

**TTS**	1	2	3	4
Xa<br>Xb	-0.0520<br>-0.0602	-0.0417<br>-0.0433	-0.0235<br>-0.0236	0.0328<br>0.0330
RDx(leftward)	0.0082	0.0016	0.0001	-0.0002
Ya<br>Yb	1.1135<br>1.2132	0.7899<br>0.8110	0.6173<br>0.6161	0.0864<br>0.0541
RDy(backward)	-0.0997	-0.0211	0.0012	0.0323
Za<br>Zb	-0.7718<br>-0.7265	-1.0294<br>-1.0164	-1.1356<br>-1.1308	-1.1132<br>-1.1089
RDz(upward)	-0.0453	-0.0130	-0.0048	-0.0043
**RD**	**0.1098**	**0.0248**	**0.0049**	**0.0326**
**TO**	1	2	3	4
Xa<br>Xb	0.1940<br>0.1852	0.3496<br>0.0905	0.4549<br>0.0150	0.6175<br>-0.3051
RDx(leftward)	0.0088	0.2591	0.4399	0.9226
Ya<br>Yb	1.3566<br>1.4312	1.2111<br>0.9993	1.1623<br>0.7951	0.9940<br>0.1586
RDy(backward)	-0.0746	0.2118	0.3672	0.8354
Za<br>Zb	-1.0733<br>-1.0191	-1.1784<br>-1.3907	-1.2059<br>-1.5236	-1.1855<br>-1.5451
RDz(upward)	-0.0542	0.2123	0.3177	0.3596
**RD**	**0.0926**	**0.3963**	**0.6552**	**1.2955**
**ITO**	1	2	3	4
Xa<br>Xb	0.0722<br>0.0635	0.1294<br>0.0845	0.1815<br>0.0573	0.2732<br>-0.1657
RDx(leftward)	0.0087	0.0449	0.1242	0.4389
Ya<br>Yb	1.2309<br>1.2898	1.0025<br>0.9148	0.9069<br>0.7148	0.6116<br>0.0899
RDy(backward)	-0.0589	0.0877	0.1921	0.5217
Za<br>Zb	-0.9930<br>-0.9401	-1.1746<br>-1.2464	-1.2332<br>-1.3868	-1.2183<br>-1.411
RDz(upward)	-0.0529	0.0718	0.1536	0.1927
**RD**	**0.0796**	**0.1219**	**0.2755**	**0.7085**

Point a is located inside the fracture line, and point b is located outside the fracture line. Xa and Xb respectively represent the displacement of the two points

relative to the origin on the X axis. Ya and Yb respectively represent the displacement of the two points on the Y axis relative to the origin. Za and Zb respectively represent the displacement of the two points on the Z axis relative to the origin.

TTS: Two transsacral screws;

TO: Triangular osteosynthesis;

ITO: Iliosacral triangular osteosynthesis

RDx: The relative displacement of the two points a, b on the X axis. Leftward is a positive value

RDy: The relative displacement of the two points a, b on the Y axis. Backward is a positive value

RDz: The relative displacement of the two points a, b on the Z axis .Upward is a positive value

RD: The total relative displacement of two points a, b in the three-dimensional direction
