# Supplementary material for: Applicable safety analysis and biomechanical study of iliosacral triangular osteosynthesis
Source: BMC Musculoskelet Disord. 2021 Nov 23;22:971. doi: 10.1186/s12891-021-04856-8 (PMC8609831; doi:10.1186/s12891-021-04856-8)
Supplement: Supplementary file 5 — Additional file 5. [file 12891_2021_4856_MOESM5_ESM.pdf]

Additional file 5

Relative displacement in left twisting

**TTS**	1	2	3	4
Xa<br>Xb	0.0037<br>-0.0069	-0.0336<br>-0.0363	-0.0411<br>-0.0420	-0.0224<br>-0.0231
RDx(leftward)	0.0106	0.0027	0.0009	0.0007
Ya<br>Yb	1.0696<br>1.1839	0.7578<br>0.7945	0.5816<br>0.5944	0.0431<br>0.0222
RDy(backward)	-0.1143	-0.0367	-0.0128	0.0209
Za<br>Zb	-0.7503<br>-0.6917	-1.0147<br>-0.9969	-1.1243<br>-1.1165	-1.1062<br>-1.0992
RDz(upward)	-0.0586	-0.0178	-0.0078	-0.0070
**RD**	**0.1289**	**0.0409**	**0.0150**	**0.0221**
**TO**	1	2	3	4
Xa<br>Xb	0.1608<br>0.1439	0.1449<br>-0.0155	0.1668<br>-0.1035	0.2270<br>-0.3451
RDx(leftward)	0.0169	0.1604	0.2703	0.5721
Ya<br>Yb	1.2240<br>1.2493	1.0098<br>0.9001	0.8975<br>0.7221	0.5564<br>0.1816
RDy(backward)	-0.0253	0.1097	0.1754	0.3748
Za<br>Zb	-0.9626<br>-0.8546	-1.1840<br>-1.1788	-1.2542<br>-1.2921	-1.2413<br>-1.3039
RDz(upward)	-0.1080	-0.0052	0.0379	0.0626
**RD**	**0.1122**	**0.1944**	**0.3244**	**0.6868**
**ITO**	1	2	3	4
Xa<br>Xb	0.1486<br>0.1394	0.0844<br>0.0640	0.0747<br>0.0112	0.0763<br>-0.1960
RDx(leftward)	0.0092	0.0204	0.0635	0.2723
Ya<br>Yb	1.1287<br>1.1888	0.9065<br>0.8504	0.7909<br>0.6634	0.4466<br>0.0921
RDy(backward)	0.0601	0.0561	0.1275	0.3545
Za<br>Zb	-0.9064<br>-0.8517	-1.1267<br>-1.1555	-1.2003<br>-1.282	-1.1941<br>-1.2995
RDz(upward)	-0.0547	0.0288	0.0817	0.1054
**RD**	**0.0818**	**0.0663**	**0.1642**	**0.4593**

Point a is located inside the fracture line, and point b is located outside the fracture

line. Xa and Xb respectively represent the displacement of the two points relative to the origin on the X axis. Ya and Yb respectively represent the displacement of the two points on the Y axis relative to the origin. Za and Zb respectively represent the displacement of the two points on the Z axis relative to the origin.

TTS: Two transsacral screws;

TO: Triangular osteosynthesis;

ITO: Iliosacral triangular osteosynthesis

RDx: The relative displacement of the two points a, b on the X axis. Leftward is a positive value

RDy: The relative displacement of the two points a, b on the Y axis. Backward is a positive value

RDz: The relative displacement of the two points a, b on the Z axis .Upward is a positive value

RD: The total relative displacement of two points a, b in the three-dimensional direction
